# Supplementary material for: Mechanisms of Antioxidant Resistance in Different Wheat Genotypes under Salt Stress and Hypoxia
Source: Int J Mol Sci. 2023 Nov 28;24(23):16878. doi: 10.3390/ijms242316878 (PMC10707134; doi:10.3390/ijms242316878)
Supplement: Supplementary file 1 [file ijms-24-16878-s001.zip › ijms-2700783-supplementary.pdf]

Table S1. Primer Information

Gene		5′-3′-sequence	length of product
MnSOD	XM_044478966.1	tga gtt tcc cgc tgt tgg at	122
		aca ggc act gtt cta tgc gt	
Cu/ZnSOD	XM_044573539.1	ctc cag tca tac tgg ttt cca tc	199
		ttc ccg agt tca cgc ttc at	
PX	XM_044466229.1	tct ctt tca caa gcc acc aag	261
		acg aag cag tcg tgg aag tg	
GPX	XM_044519104.1	gca aag tat ccc cgt cgtc ga	184
		gta cct cga caa aag cac cc	
CAT	NM_001405704.1	cgc att gtc gtc gta cgt cat tc	217
		ctc aga cat agc gac gct cc	
GST	AY377972.1	cag gca gtc aat cct cgg ac	174
		agc ttt cat ttt gat atc ttt ggg c	
